# Killer Immunoglobulin-Like Receptor **(**KIR**)** Centromeric-AA Haplotype Is Associated with Ethnicity and Tuberculosis Disease in a Canadian First Nations Cohort

**DOI:** 10.1371/journal.pone.0067842

**Published:** 2013-07-04

**Authors:** Kali Braun, Linda Larcombe, Pamela Orr, Peter Nickerson, Joyce Wolfe, Meenu Sharma

**Affiliations:** 1 Department of Medical Microbiology, University of Manitoba, Winnipeg, Manitoba, Canada; 2 Department of Internal Medicine, University of Manitoba, Winnipeg, Manitoba, Canada; 3 National Reference Centre for Mycobacteriology, Public Health Agency of Canada, Winnipeg, Manitoba, Canada; Karolinska Institutet, Sweden

## Abstract

Killer immunoglobulin-like receptors (KIR) on natural killer (NK) cells interact with other immune cells to monitor the immune system and combat infectious diseases, such as tuberculosis (TB). The balance of activating and inhibiting KIR interactions helps determine the NK cell response. In order to examine the enrichment or depletion of KIRs as well as to explore the association between TB status and inhibitory/stimulatory KIR haplotypes, we performed KIR genotyping on samples from 93 Canadian First Nations (Dene, Cree, and Ojibwa) individuals from Manitoba with active, latent, or no TB infection, and 75 uninfected Caucasian controls. There were significant differences in KIR genes between Caucasians and First Nations samples and also between the First Nations ethnocultural groups (Dene, Cree, and Ojibwa). When analyzing ethnicity and tuberculosis status in the study population, it appears that the KIR profile and centromeric haplotype are more predictive than the presence or absence of individual genes. Specifically, the decreased presence of haplotype B centromeric genes and increased presence of centromeric-AA haplotypes in First Nations may contribute to an inhibitory immune profile, explaining the high rates of TB in this population.

## Introduction

Natural killer (NK) cells bridge the innate and adaptive immune response to infection by the production of cytokines [1]. The activity of NK cells is controlled by a balance of inhibitory and stimulatory signals generated when a ligand binds to a killer immunoglobulin-like receptor (KIR) on the NK cell surface. The interaction of KIR and self-human leukocyte antigen (HLA) class I allows NK cells to identify and inhibit immune responses to normally functioning cells. Inhibitory KIRs contain an immunoreceptor tyrosine-based inhibition motif (ITIM) which interacts with a phosphatase, preventing phosphorylation of the activation cascade (NK cell cytotoxicity and cytokine release). Activating KIRs lack ITIMs but interact with a signalling adaptor that contains an immunoreceptor tyrosine-based activation motif (ITAM) that interacts with a kinase, allowing progression of the activation cascade [2]. When the stimulatory interactions overcome the inhibitory interactions, the outcome is NK cell cytotoxicity resulting in cytokine release [1], which plays a significant role in the immune response to infectious diseases such as tuberculosis (TB) [3].

KIR genes are highly variable in nature due to both polygenic and multi-allelic polymorphisms [1]. KIR genes have a high level of sequence similarity leading to a predisposition for homologous recombination, explaining the expansion and contraction of the KIR locus [4]. Genetic susceptibility or resistance to infectious disease has been highly correlated with ethnicity, which in conjunction with host risk factors, can determine disease progression [5]–[8]. The KIR diversity, as well as activating/inhibiting balance of KIR genes, contributes to distinct disease outcomes between ethnic populations. For example, KIR2DL3 has been found to be significantly more prevalent in Lebanese and Mexican TB patients compared to control populations without TB [9], [10]. Distinct outcomes of immune-regulated diseases are primarily due to differential expression of cytokines between different populations such as Caucasians, First Nations, and other ethnicities.

Tuberculosis is caused by infection with the bacterium *Mycobacterium tuberculosis*, spread by airborne particles generated by an infectious person [11]. It is the inability of the infected macrophage to contain the *M. tuberculosis* that is fundamental to the pathogenesis of TB. Approximately 90% of infected non-immunosuppressed individuals never develop active disease, while up to 10% may develop disease at some point during their lifetime [11], [12]. Latent TB infection (LTBI) refers to the condition in which *M. tuberculosis* remains viable in the macrophage but retains only a small amount of metabolic activity [13]. LTBI has historically been captured as exposed or unexposed, as compared to a gradient or degree of exposure [14]. Current evidence suggests LTBI may be better explained as a spectrum of disease correlating to degree, duration, and proximity of exposure [14].

The World Health Organization estimated the global prevalence of LTBI at 33%, with 9.2 million new cases of TB in 2010 (128/100,000 population) [15]. In the same year, 1.1 million people died of TB. Canada reported 1577 new active cases of TB (4.6/100,000) in 2010 [16]. The incidence of TB in Manitoba in 2010 was 10.7/100,000, with a disproportionate incidence in Canadian born Aboriginal (First Nations, Metis, and Inuit) peoples (39.8/100,000) compared with Canadian born non-Aboriginal peoples (1.6/100,000) [16]. The overall incidence of TB on First Nations reserve communities (including those of the Dene, Cree, and Ojibwa ethnocultural groups) in the Canadian province of Manitoba reached 58.3/100,000 in 2010 [16]. Rates of TB in certain Manitoba First Nations communities exceeds 400/100,000 [17]–[19]. The determinants of TB in Canadian First Nations peoples include those associated with host virulence [17], [20], host susceptibility [21], [22] and social/environmental factors [23].

The discovery of an unexpected level of diversity within the KIR genes has led to a search for their role in human disease [4]. The presence or absence of KIR genes may be associated with tuberculosis status (active disease, latent disease, uninfected) as well as ethnicity of an individual [9], [10], [24]. It is hypothesized that the differences in genetic KIR profiles between Manitoba First Nations and Caucasian individuals elicits differential cytokine expression and eventually contributes to the outcome to TB infection. Identification of ethnic specific genes that confer susceptibility to TB infection will help to better understand the interaction of host genetics and the immune system. In this study we examined the enrichment or depletion of KIR genes in Manitoba First Nations and Caucasian populations with special focus on First Nations with active, latent, or uninfected TB status. In addition, we sought to explore the association between TB status and inhibitory/stimulatory KIR profiles and haplotypes.

## Materials and Methods

### Study Populations

The 168 samples consisted of whole blood and/or buccal swabs from adult Dene (n = 63), Cree (n = 19), and Ojibwa (n = 11) individuals from three Northern Manitoba First Nations communities, as well as uninfected Caucasian controls (n = 75) from Winnipeg, Manitoba. Within each First Nations groups, there were internal uninfected controls. The samples were obtained with informed written consent, in addition to approval by the University of Manitoba Ethics Board and the First Nations communities involved. Interviews and questionnaires were conducted probing for evidence of investigation and treatment of active or latent tuberculosis, including history of tuberculin skin test (TST) and treatment regimens. The study comprised of 59 First Nations individuals with no TB, 14 individuals with latent TB, and 20 individuals with active TB. Sample demographics can be seen in Table 1. Although most First Nations individuals involved received bacilli Calmette-Guérin (BCG) vaccination at birth, the interval of greater than 18 years between BCG and TST prevents any significant number of false-positives due to cross-reactivity [25], [26]. Therefore history of a positive TST result was assumed to be from a delayed-type hypersensitivity reaction to the tuberculin, and not a cross reaction from potential previous BCG vaccination.

**Table 1:** Study population demographics.

Parameter	Value	Number of Isolates (%)			
		Caucasian	Dene	Cree	Ojibwa
		n = 75	n = 63	n = 19	n = 11
Gender	Male	25 (33.3)	34 (54.0)	8 (42.1)	6 (54.5)
	Female	50 (67.7)	29 (46.0)	11 (57.9)	5 (45.5)
Age	≤19	0 (0.0)	3 (4.8)	0 (0.0)	0 (0.0)
	20–39	0 (0.0)	19 (30.1)	1 (5.3)	2 (18.2)
	40–59	44 (58.7)	24 (38.1)	14 (73.7)	6 (54.5)
	≥60	31 (41.3)	17 (27.0)	5 (26.3)	3 (27.3)
Disease Status	No TB	75 (100.0)	38 (60.3)	11 (57.9)	10 (90.9)
	Latent	-	10 (15.9)	4 (21.1)	0 (0.0)
	Active	-	15 (23.8)	4 (21.1)	1 (9.1)

### DNA Extraction and Replication

Genomic DNA was extracted using Qiagen DNA Mini Kit as per manufacturer's instructions (Qiagen, Louisville, KY). Genomic DNA was eluted from the silica-membrane-based nucleic acid purification column with 200 μL of elution buffer containing 10 mM Tris-Cl and 0.5 mM EDTA, pH 9.0, and stored at −20°C until analysis. The samples were subjected to whole genome replication using the Qiagen Repli-G mini kit as per manufacturer's instructions to increase DNA concentration of the testing sample.

### KIR Genotyping

The concentration of DNA was normalized to 100 μg/mL at 260 nm using the SmartSpec Plus spectrophotometer (Bio-Rad, Mississauga, ON). KIR genotyping was performed by sequence-specific primer polymerase chain reaction (SSP-PCR) using the Miltenyi Biotec KIR typing kit (Auburn, CA) with the following adjustments: the PCR denaturation step was extended from one minute to two minutes in order to ensure proper amplification of the internal beta-actin control in each reaction well. Additionally, TAE buffer was replaced by TBE buffer for gel electrophoresis. The kit tested for the presence or absence of the following genes: KIR2DL1, KIR2DL2, KIR2DL3, KIR2DL4, KIR2DL5all (A and/or B), KIR2DL5A, KIR2DL5B, KIR2DS1, KIR2DS2, KIR2DS3, KIR2DS4del (KIR1D), KIR2DS4ins (full length), KIR2DS5, KIR3DL1, KIR3DL2, KIR3DL3, KIR3DS1, KIR2DP1, and KIR3DP1. The amplicons were visualized with UV light (Bio-Rad Gel Doc EZ Imager, Mississauga, ON) following gel electrophoresis at 13V/cm on a 2% agarose gel containing ethidium bromide. Proficiency testing was performed using samples with known KIR genotype from the Fred Hutchinson Cancer Research Center, International Histocompatibility Working Group Cell and Gene Bank (Seattle, WA).

### Statistical Analysis

The presence (1) or absence (0) of a gene was assigned a binary code and this data was entered into BioNumerics software version 5.0 (Applied Maths, Belgium). Data for each individual's KIR genes were combined into a KIR profile and clustered to identify prevalent profiles among specified groups using the categorical co-efficient and UPGMA [27]. KIR gene frequencies were tabulated by direct counts from the clustered profiles to determine frequency within a defined group. Differences between Caucasians, First Nations, First Nations subsets and TB status groups were estimated using the two-tailed Fisher's exact test (GraphPad Software, La Jolla, CA). Haplotype designation was determined as previously described [28], [29].

## Results and Discussion

**Figure 1:**
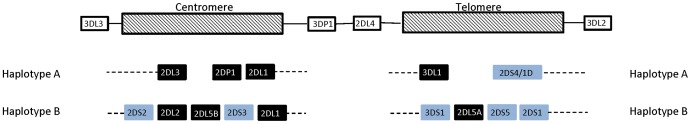
Schematic of KIR gene haplotypes A and B. white – framework genes, grey – activating KIR, black – inhibitory KIR; note that KIR2DP1 and KIR3DP1 are pseudogenes, and that KIR2DL2/2DL3 as well as KIR3DL1/3DS1 represent the same locus.

All samples consistently contained the framework genes KIR2DL4, KIR3DL2, KIR3DL3, and the pseudogenes KIR2DP1 and KIR3DP1, as expected [28]. There were many significant differences (P-value ≤0.05) in the frequency of KIR genes between Caucasians and First Nations. Differences were seen in KIR2DL2, KIR2DL5A, KIR2DS1, KIR2DS2, KIR2DS3, KIR1D, KIR2DS4, KIR2DS5, KIR3DL1, and KIR3DS1 ( Table S1). More specifically, compared to Caucasians, significant differences were seen in gene frequencies in Dene First Nations (KIR2DL2, KIR2DS2, KIR2DS3, KIR1D, KIR2DS4, KIR2DS5, KIR3DL1), Cree First Nations (KIR2DL2, KIR2DL5all, KIR2DL5A, KIR2DL5B, KIR2DS1, KIR2DS2, KIR1D, KIR2DS5, KIR3DL1, KIR3DS1), and Ojibwa First Nations (KIR2DL1, KIR2DS5). We found that haplotype B centromeric genes (KIR2DL2, KIR2DS2, KIR2DS3) were reduced while haplotype B telomeric genes (KIR3DS1, KIR2DL5A, KIR2DS1, KIR2DS5) were more prominent in Manitoba First Nations when compared with Caucasians. These findings agreed with recent work by Rempel, *et al* [30]. Additionally, haplotype A telomeric genes KIR2DS4 and KIR3DL1 were significantly increased, and decreased, respectively in First Nations compared to Caucasians. Haplotypes A/B as well as centromeric/telomeric distinction is illustrated in Figure 1. These additional findings, along with the haplotype analysis (see below) support the view that First Nations individuals have a stronger inhibitory phenotype compared to Caucasians. This conclusion is the opposite proposed by the literature, and the difference may be attributed to different ethnocultural sample populations: Rempel, *et al* study contained 70% Oji-Cree First Nations compared to 68% Dene First Nations in our study.

The presence of KIR1D was significantly lower in First Nations (both Dene and Cree) individuals. KIR1D is an allele of KIR2DS4, with a 22 base pair deletion resulting in a truncated protein. The loss of a transmembrane domain as well as a cytoplasmic domain in KIR1D leads to a protein that is not anchored to the membrane [28], [31]. When comparing KIR gene frequencies in First Nations individuals with TB status (active and latent) to those with negative TB status, the only gene approaching statistical significance was KIR1D, found in 45.76% of First Nations with no disease but only 26.47% in First Nations with TB status (p-value 0.0795; Table S2). Few disease association studies [32]–[34] have been performed to date on KIR1D, none focusing on tuberculosis. Since KIR1D cannot anchor to the membrane, it becomes a secreted KIR molecule. It has been hypothesized that there may be a role for a soluble KIR to act as a ligand for an unidentified receptor, or to “mop up” soluble HLA, which could interfere with NK cell function [31], [34].

**Figure 2:**
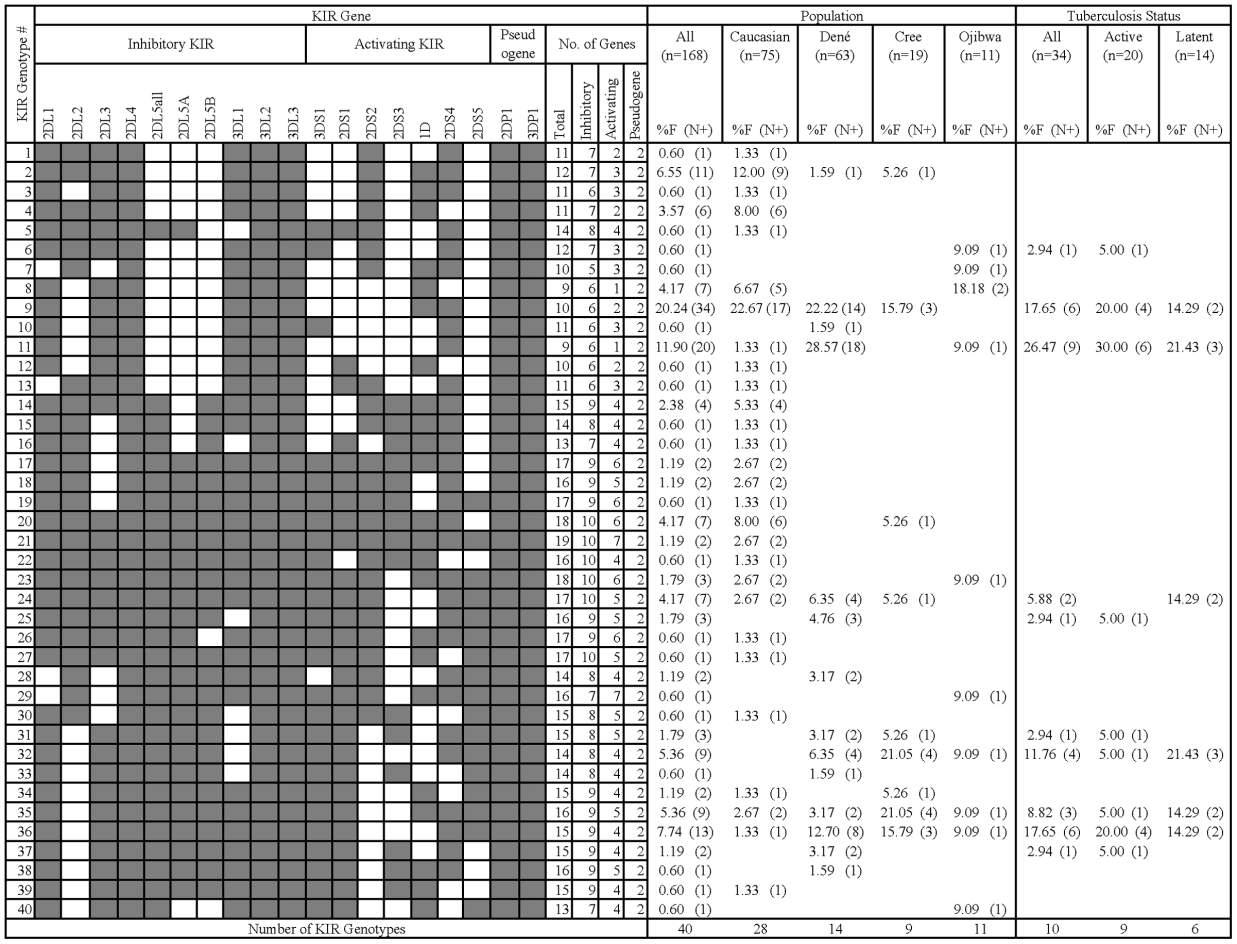
Frequency of KIR genotypes in human populations. Forty distinct KIR types were seen in these 168 individuals that differ from each other by the presence of (shaded box) or absence (white box) of 19 KIR genes (KIR2DL5 broken down into 2DL5A, 2DL5B, and 2DL5all; KIR2DS4 broken down into 1D and 2DS4). Frequency (%F) of each genotype is expressed as a percentage and is defined as the number of individuals having the genotype (N+) divided by the number of individuals (n) in the population or tuberculosis status group.

For those with KIR2DS4 as their only activating gene, the full length KIR2DS4 was more common than KIR1D, as anticipated [31]. Of the forty different profiles identified in our 168 samples, only seven individuals (4.2%; genotype #8) had KIR1D as their only activating KIR compared to 20 individuals (11.9%; genotype #11) with KIR2DS4 as their only activating KIR (Figure 2). Those individuals with KIR1D as their only “activating” KIR (KIR1D^+/+^), do not have any intact activating membrane-anchored KIR due to the loss of the transmembrane domain. These individuals all belonged to the no disease group.

First Nations were significantly more likely to have AA-AB and AA-BB haplotypes, and less likely to have AB-AA and AB-AB haplotypes than Caucasians (Table 2). In addition, significantly more First Nations individuals with TB status (30/34, 88.2%) were found to have a centromeric-AA haplotype (P-value <0.0001). Half of the active cases of tuberculosis were of the haplotype AA-AA, which contains the fewest number of activating genes of all haplotypes. It may be speculated that the lack of activating KIR genes leads to a worsened immune response against tuberculosis infection.

**Table 2:** Frequency of centromeric and telomeric haplotypes in Caucasians and First Nations.

Haplotype		Ethnicity			Tuberculosis Status in First Nations		
Centromeric	Telomeric	Caucasian	First Nations		Latent	Active	Uninfected
		(n = 75)	(n = 93)	P-value	(n = 14)	(n = 20)	(n = 59)
AA	AA	25 (33.3)	38 (40.9)	0.3399	5 (35.7)	10 (50.0)	23 (39.0)
AA	AB	5 (6.7)	25 (26.9)	**0.0006**	4 (28.6)	6 (30.0)	15 (25.4)
AB	AA	21 (28.0)	2 (2.2)	**<0.0001**			2 (3.4)
AB	AB	15 (20.0)	8 (8.6)	**0.0421**	2 (14.3)	1 (5.0)	5 (8.5)
AA	BB	0 (0.0)	13 (14.0)	**0.0006**	3 (21.4)	2 (10.0)	8 (13.6)
AB	BB	1 (1.3)	3 (3.2)	0.6296		1 (5.0)	2 (3.4)
BB	AA	1 (1.3)	3 (3.2)	0.6296			3 (5.1)
BB	AB	5 (6.7)	1 (1.1)	0.0900			1 (1.7)
BB	BB	1 (1.3)	0 (0.0)	0.4464			
BB	−/−	1 (1.3)	0 (0.0)	0.4464			

Significant P-values (≤0.05) are bolded.

In summary, there are significant differences in KIR genes between Caucasians and First Nations participants in this study, and between the participants of different First Nation ethnocultural groups (Dene, Cree, and Ojibwa). When looking at tuberculosis status, it appears that the KIR profile and centromeric haplotype are more predictive than the presence or absence of individual genes. The increased presence of all centromeric-AA haplotypes in First Nations (81.8%) compared to Caucasian (40.0%) participants, along with the overwhelming amount of TB (88.2%) in these same haplotypes, indicates a predictive relationship between KIR, ethnicity, and disease.

When assessing study validity, results from these First Nations communities are not necessarily generalizable to all First Nations communities in the province of Manitoba. The small number of samples also affects the internal validly, however the degree of participation is not surprising in the context of research performed in Canadian Aboriginal populations in remote communities [35]. It is because of this that the statistical power of the comparisons was limited.

The sampling of additional individuals is needed to confirm the generalizability of these findings to the larger provincial Aboriginal populations. In addition, further work, such as sequence analysis of select genetic regions, needs to be done to further clarify the relationship between infectious diseases and KIR in individuals with tuberculosis.

## Supporting Information

Table S1 Killer immunoglobulin-like receptor (KIR) gene frequencies in First Nations and Caucasians.
(XLSX)

Table S2 Killer immunoglobulin-like receptor (KIR) gene frequencies in First Nations by tuberculosis status.
(XLSX)

## References

[1] Martin MP, Carrington M (2008) KIR locus polymorphisms: genotyping and disease association analysis. Methods Mol Biol 415: 49–64 doi:_10.1007/978-1-59745-570-1_3 18370147 10.1007/978-1-59745-570-1_3 PMC10763752

[2] Kelley J, Walter L, Trowsdale J (2005) Comparative genomics of natural killer cell receptor gene clusters. PLoS Genet 1: 129–139 doi:10.1371/journal.pgen.0010027 16132082 10.1371/journal.pgen.0010027 PMC1193534

[3] Carrington M, Norman P (2003) The KIR Gene Cluster. Bethesda, MD: National Centre for Biotechnology Information.

[4] Khakoo SI, Carrington M (2006) KIR and disease: a model system or system of models? Immunol Rev 214: 186–201 doi:10.1111/j.1600-065X.2006.00459.x 17100885 10.1111/j.1600-065X.2006.00459.x

[5] Delgado JC, Baena A, Thim S, Goldfeld AE (2002) Ethnic – Specific Genetic Associations with Pulmonary Tuberculosis. The Journal of Infectious Diseases 186: 1463–1468 doi:10.1086/344891 12404162 10.1086/344891

[6] Middleton D, Gonzelez F (2010) The extensive polymorphism of KIR genes. Immunology 129: 8–19 doi:10.1111/j.1365-2567.2009.03208.x 20028428 10.1111/j.1365-2567.2009.03208.x PMC2807482

[7] Morris GAJ, Edwards DRV, Hill PC, Wejse C, Bisseye C, et al (2011) Interleukin 12B (IL12B) genetic variation and pulmonary tuberculosis: a study of cohorts from The Gambia, Guinea-Bissau, United States and Argentina. PLoS ONE 6: e16656 doi:10.1371/journal.pone.0016656 21339808 10.1371/journal.pone.0016656 PMC3037276

[8] La D, Czarnecki C, El-Gabalawy H, Kumar A, Meyers AFA, et al (2011) Enrichment of variations in KIR3DL1/S1 and KIR2DL2/L3 among H1N1/09 ICU patients: an exploratory study. PLoS ONE 6: e29200 doi:10.1371/journal.pone.0029200 22216211 10.1371/journal.pone.0029200 PMC3247251

[9] Mahfouz R, Halas H, Hoteit R, Saadeh M, Shamseddeen W, et al (2011) Study of KIR genes in Lebanese patients with tuberculosis. Int J Tuberc Lung Dis 15: 1688–1691 doi:10.5588/ijtld.11.0138 22118180 10.5588/ijtld.11.0138

[10] Méndez A, Granda H, Meenagh A, Contreras S, Zavaleta R, et al (2006) Study of KIR genes in tuberculosis patients. Tissue Antigens 68: 386–389 doi:10.1111/j.1399-0039.2006.00685.x 17092251 10.1111/j.1399-0039.2006.00685.x

[11] Versalovic J (n.d.) Manual of Clinical Microbiology. 10th ed. Washington, DC: ASM Press.

[12] Public Health Agency of Canada, Canadian Lung Assocation/Canadian Thoracic Society (2007) Canadian Tuberculosis Standards. 6th ed. Canada: Minister of Health.

[13] Vernon A (2013) Treatment of latent tuberculosis infection. Semin Respir Crit Care Med 34: 67–86 doi:10.1055/s-0032-1333544 23460007 10.1055/s-0032-1333544

[14] Trajman A, Steffen RE, Menzies D (2013) Interferon-Gamma Release Assays versus Tuberculin Skin Testing for the Diagnosis of Latent Tuberculosis Infection: An Overview of the Evidence. Pulm Med 2013: 601737 doi:10.1155/2013/601737 23476763 10.1155/2013/601737 PMC3582085

[15] World Health Organization (2011) Global Tuberculosis Control 2011. Geneva, Switzerland: WHO Press.

[16] Tuberculosis in Canada 2010, Pre-Release – Public Health Agency of Canada (n.d.). Available: http://www.phac-aspc.gc.ca/tbpc-latb/pubs/tbcan10pre/index-eng.php. Accessed 2012 May 16.

[17] Petrelli D, Kaushal Sharma M, Wolfe J, Al-Azem A, Hershfield E, et al (2004) Strain-related virulence of the dominant Mycobacterium tuberculosis strain in the Canadian province of Manitoba. Tuberculosis (Edinb) 84: 317–326 doi:10.1016/j.tube.2004.01.001 15207807 10.1016/j.tube.2004.01.001

[18] Sharma MK, Al-Azem A, Wolfe J, Hershfield E, Kabani A (2003) Identification of a predominant isolate of Mycobacterium tuberculosis using molecular and clinical epidemiology tools and in vitro cytokine responses. BMC Infect Dis 3: 3. 12697047 10.1186/1471-2334-3-3 PMC154093

[19] First Nations Regional Health Survey (RHS) 2008-10 – National Report.pdf (n.d.). Available: http://www.fnigc.ca/sites/default/files/First%20Nations%20Regional%20Health%20Survey%20(RHS)%202008-10%20-%20National%20Report.pdf. Accessed 2012 Sep 25.

[20] Arvanitakis Z, Long RL, Hershfield ES, Manfreda J, Kabani A, et al (1998) M. tuberculosis molecular variation in CNS infection: evidence for strain-dependent neurovirulence. Neurology 50: 1827–1832. 9633735 10.1212/wnl.50.6.1827

[21] Larcombe LA, Orr PH, Lodge AM, Brown JS, Dembinski IJ, et al (2008) Functional gene polymorphisms in canadian aboriginal populations with high rates of tuberculosis. J Infect Dis 198: 1175–1179 doi:10.1086/592049 18713057 10.1086/592049

[22] Larcombe L, Rempel JD, Dembinski I, Tinckam K, Rigatto C, et al (2005) Differential cytokine genotype frequencies among Canadian Aboriginal and Caucasian populations. Genes Immun 6: 140–144 doi:10.1038/sj.gene.6364157 15674369 10.1038/sj.gene.6364157

[23] Larcombe L, Nickerson P, Singer M, Robson R, Dantouze J, et al (2011) Housing conditions in 2 Canadian First Nations communities. Int J Circumpolar Health 70: 141–153. 21524357 10.3402/ijch.v70i2.17806

[24] Shahsavar F, Mousavi T, Azargon A, Entezami K (2012) Association of KIR3DS1+HLA-B Bw4Ile80 Combination with Susceptibility to Tuberculosis in Lur Population of Iran. Iran J Immunol 9: 39–47 doi:IJIv9i1A3 22426166 10.22034/iji.2012.16855

[25] Farhat M, Greenaway C, Pai M, Menzies D (2006) False-positive tuberculin skin tests: what is the absolute effect of BCG and non-tuberculous mycobacteria? Int J Tuberc Lung Dis 10: 1192–1204. 17131776

[26] Reid JK, Ward H, Marciniuk D, Hudson S, Smith P, et al (2007) The effect of neonatal bacille Calmette-Guérin vaccination on purified protein derivative skin test results in Canadian aboriginal children. Chest 131: 1806–1810 doi:10.1378/chest.06-1133 17400666 10.1378/chest.06-1133

[27] Clifford HT (1975) An Introduction to Numerical Classification. Academic Press.

[28] Hsu KC, Liu X-R, Selvakumar A, Mickelson E, O'Reilly RJ, et al (2002) Killer Ig-like receptor haplotype analysis by gene content: evidence for genomic diversity with a minimum of six basic framework haplotypes, each with multiple subsets. J Immunol 169: 5118–5129. 12391228 10.4049/jimmunol.169.9.5118

[29] Gutiérrez-Rodríguez ME, Sandoval-Ramírez L, Díaz-Flores M, Marsh SGE, Valladares-Salgado A, et al (2006) KIR gene in ethnic and Mestizo populations from Mexico. Hum Immunol 67: 85–93 doi:10.1016/j.humimm.2005.11.007 16698429 10.1016/j.humimm.2005.11.007

[30] Rempel JD, Hawkins K, Lande E, Nickerson P (2011) The potential influence of KIR cluster profiles on disease patterns of Canadian Aboriginals and other indigenous peoples of the Americas. Eur J Hum Genet 19: 1276–1280 doi:10.1038/ejhg.2011.114 21731058 10.1038/ejhg.2011.114 PMC3230357

[31] Middleton D, Gonzalez A, Gilmore PM (2007) Studies on the expression of the deleted KIR2DS4*003 gene product and distribution of KIR2DS4 deleted and nondeleted versions in different populations. Hum Immunol 68: 128–134 doi:10.1016/j.humimm.2006.12.007 17321903 10.1016/j.humimm.2006.12.007

[32] Giebel S, Nowak I, Wojnar J, Krawczyk-Kulis M, Holowiecki J, et al.. (2008) Association of KIR2DS4 and its variant KIR1D with leukemia. Leukemia 22: 2129–2130; discussion 2130–2131. doi:10.1038/leu.2008.108. 10.1038/leu.2008.108 18463675

[33] Zhang Y, Wang B, Ye S, Liu S, Liu M, et al (2010) Killer cell immunoglobulin-like receptor gene polymorphisms in patients with leukemia: possible association with susceptibility to the disease. Leuk Res 34: 55–58 doi:10.1016/j.leukres.2009.04.022 19450876 10.1016/j.leukres.2009.04.022

[34] Zhuang Y-L, Zhu C-F, Zhang Y, Song Y-H, Wang D-J, et al (2012) Association of KIR2DS4 and its variant KIR1D with syphilis in a Chinese Han population. Int J Immunogenet 39: 114–118 doi:10.1111/j.1744-313X.2011.01063.x 22128817 10.1111/j.1744-313X.2011.01063.x

[35] Wilson S (2008) Research is Ceremony: Indigenous Research methods. Winnipeg: Fernwood Publishing. 144 p.

